# A Transcriptome-Led Exploration of Molecular Mechanisms Regulating Somatostatin-Producing D-Cells in the Gastric Epithelium

**DOI:** 10.1210/en.2015-1301

**Published:** 2015-08-04

**Authors:** Alice Adriaenssens, Brian Yee Hong Lam, Lawrence Billing, Katie Skeffington, Sabine Sewing, Frank Reimann, Fiona Gribble

**Affiliations:** Metabolic Research Laboratories (A.A., B.Y.H.L., L.B., K.S., F.R., F.G.), Wellcome Trust-Medical Research Council Institute of Metabolic Science, Addenbrooke's Hospital, Cambridge CB2 0QQ, United Kingdom; and Pharma Research and Early Development (S.S.), Roche Innovation Center Basel, F. Hoffmann-La Roche AG, 4070 Basel, Switzerland

## Abstract

The stomach epithelium contains a myriad of enteroendocrine cells that modulate a range of physiological functions, including postprandial secretion of regulatory peptides, gastric motility, and nutrient absorption. Somatostatin (SST)-producing D-cells are present in the oxyntic and pyloric regions of the stomach, and provide a tonic inhibitory tone that regulates activity of neighboring enteroendocrine cells and gastric acid secretion. Cellular mechanisms underlying the effects of regulatory factors on gastric D-cells are poorly defined due to problems in identifying primary D-cells, and uncertainty remains about which stimuli influence D-cells directly. In this study, we introduce a transgenic mouse line, SST-Cre, which upon crossing with Cre reporter strains, facilitates the identification and purification of gastric D-cells, or cell-specific expression of genetically encoded calcium indicators. Populations of D-cells from the gastric antrum and corpus were isolated and analyzed by RNA sequencing and quantitative RT-PCR. The expression of hormones, hormone receptors, neurotransmitter receptors, and nutrient receptors was quantified. *Pyy*, *Gipr*, *Chrm4*, *Calcrl*, *Taar1*, and *Casr* were identified as genes that are highly enriched in D-cells compared with SST-negative cells. Hormone secretion assays performed in mixed gastric epithelial cultures confirmed that SST secretion is regulated by incretin hormones, cholecystokinin, acetylcholine, vasoactive intestinal polypeptide, calcitonin gene-related polypeptide, oligopetides, and trace amines. Cholecystokinin and oligopeptides elicited increases in intracellular calcium in single-cell imaging experiments performed using cultured D-cells. Our data provide the first transcriptomic analysis and functional characterization of gastric D-cells, and identify regulatory pathways that underlie the direct detection of stimuli by this cell type.

The enteroendocrine system of the gastrointestinal (GI) tract is recognized to be the largest endocrine organ in the body. Composed of varying types of enteroendocrine cells (EECs) working in concert, it plays a major role in mediating postprandial secretion of regulatory peptides, gastric motility, and nutrient absorption (1). Due to their position in the mucosa of the GI tract, EECs are in a prime location for relaying the composition of luminal contents locally and to other areas of the body through a range of paracrine and endocrine signals. The somatostatin (SST)-producing D-cell is an EEC of particular interest due to the profound inhibition exerted by SST over other EECs (2), highlighting D-cells as critical modulators of the enteroendocrine axis.

Although produced in various areas of the body, including the hypothalamus, pancreas, and nerve fibers of the GI tract, the major site of SST production is gut mucosal D-cells (3, 4). The tonic inhibitory tone provided by D-cells is known to regulate smooth muscle contractility, nutrient absorption, and the secretion of key regulatory hormones (5–9). In the stomach, the main site of SST production in the gut, a primary role of SST is to regulate intragastric pH via restricting gastric acid secretion (2). Located in both the oxyntic and pyloric glands of the stomach mucosa, D-cells possess cytoplasmic extensions containing secretory vesicles that terminate near gastrin, parietal, and enterochromaffin-like cells, allowing D-cells directly to inhibit the release of gastrin, gastric acid and histamine, respectively (10–12). This inhibition is believed to be mediated largely via binding to the G_i_-coupled SST receptor 2 on target cells (13).

Ultrastructural analyses have revealed that most D-cells in the gastric corpus and antrum are open type (14), allowing them to make direct contact with, and potentially sense the composition of, the luminal contents. The oral ingestion of carbohydrate and the digestion products of fat and protein have been shown to stimulate SST release (15–17). Gut perfusion studies further showed that the luminal presence of nutrients in the stomach is key to SST secretion (18), suggesting that direct chemosensation of foodstuffs provides an important mechanism by which D-cells respond to changes in nutritive status, and act to adjust luminal pH accordingly.

In addition to nutrient-based secretagogues, SST release from the stomach is controlled by the vagus nerve and various enteric nervous system (ENS) neurotransmitters. SST is persistently released between meals to suppress interprandial acid secretion (2, 8). Activation of the efferent vagus upon food ingestion inhibits SST release, via a mechanism proposed to involve muscarinic M_2_ and M_4_ receptors expressed on D-cells (19), thereby releasing the brake on gastrin, histamine and acid secretion (20, 21). Towards the end of a meal, SST release is reinitiated, switching off gastric acid secretion. Peptides produced by the ENS that have been reported to stimulate SST release include vasoactive intestinal polypeptide (VIP), calcitonin gene-related polypeptide (CGRP), and pituitary adenylate cyclase-activating peptide (PACAP) (22–24). Hormonal signals from the small intestine and stomach, such as glucagon-like peptide-1 (GLP-1), glucose-dependent insulinotropic polypeptide (GIP) (also known as gastric inhibitory polypeptide), cholecystokinin (CCK), and gastrin, acting in addition to luminal signals such as pH, have also been implicated in this mechanism (2, 25–29).

Although previous studies have identified a number of hormonal, neural, and luminal signals that regulate SST secretion, most laboratories have worked with intact animals, whole perfused stomachs or gastric tissue pieces, and in these systems it is difficult to distinguish which stimuli act directly on D-cells and which exert indirect effects mediated by the local ENS or endocrine cells. The objective of this study was to investigate which of the proposed mediators of SST release act through direct interaction with D-cells. We developed a bacterial artificial chromosome transgenic mouse model, SST-Cre, in which the *Sst* promoter drives expression of Cre-recombinase. When crossed with fluorescent reporter strains such as ROSA26-enhanced yellow fluorescent protein (EYFP) and ROSA26-GCaMP3, fluorescent labeling and intracellular calcium sensor expression were restricted to the D-cell population. By fluorescence-activated cell sorting (FACS) purification and RNA sequencing analysis of D-cells, we identified which stimuli are associated with the expression of matching G protein-coupled receptors (GPCRs). We used primary cultures of murine gastric epithelium, in which neural connections have been disrupted, to assess the impact of different candidate stimuli on SST secretion. Relevant stimuli were also tested in single-cell, real-time calcium imaging experiments. Here, we present the first transcriptomic characterization of murine gastric D-cells and the elucidation of cellular machinery responsible for the meal-dependent regulation of SST secretion from the stomach.

## Materials and Methods

### Solutions

Unless otherwise stated, all chemicals were obtained from Sigma-Aldrich. The standard bath solution contained 138 mmol/L NaCl, 4.5 mmol/L KCl, 4.2 mmol/L NaHCO_3_, 1.2 mmol/L NaH_2_PO_4_, 2.6 mmol/L CaCl_2_, 1.2 mmol/L MgCl_2_, and 10 mmol/L HEPES (pH 7.4; NaOH). Test reagents were prepared as 1000× stocks. CCK, GLP-1, peptide YY (PYY) (all Tocris Bioscience), CGRP, acetylcholine, and gastrin were made up in molecular biology grade water. VIP and bombesin (Tocris Bioscience) were prepared in 1% (vol/vol) acetic acid. NPS2143-HCl (Tocris Bioscience) and Ro5166017 (Roche) were dissolved in dimethyl sulfoxide. These stock solutions were diluted to the final concentrations indicated. Peptone was prepared as a 0.5% (wt/vol) solution in standard bath solution.

### Animals

Animal procedures were approved by the local ethics committee and conformed to the United Kingdom Home Office regulations. Mice were killed via cervical dislocation, and stomachs were collected into ice-cold Leibovitz-15 medium.

A detailed description of the generation of mice expressing Cre-recombinase under the control of the *Sst* promoter is given in the Supplemental Methods and Supplemental Figure 1A. In brief, we made transgenic mice using a Nonobese diabetic-mouse derived bacterial artificial chromosome in which the coding sequence for *Sst* was replaced while retaining approximately 118-kbp 5′ and approximately 98-kbp 3′ of the *Sst*-start and *Sst*-stop codons, respectively. SST-Cre mice were crossed with ROSA26-EYFP, ROSA26-tandem red fluorescent protein (tdRFP), and ROSA26-GCaMP3 mice (30, 31), as indicated to produce double or triple transgenic mouse lines. Male and female mice were used interchangeably, in approximately equal numbers.

### Immunohistochemistry

Freshly isolated mouse stomach or pancreas was fixed in 4% (wt/vol) paraformaldehyde for 24 hours at 4°C and cryoprotected via sucrose gradient, starting with 15% sucrose for 6 hours at room temperature and finishing with 30% sucrose for 24 hours at 4°C. The tissue was then embedded in optimum cutting temperature compound before slicing. Tissue sections (7 μm) were blocked in 10% donkey serum, 0.05% Tween 80 for 1 hour at room temperature. Sections were then incubated with primary antibodies against SST (1:1000; Dako) and green fluorescent protein (GFP) (1:1000; Abcam) overnight at room temperature (for antibodies, see Table 1). Sections were rinsed with 5% donkey serum, 0.05% Tween 80 before being incubated for 1 hour at room temperature with Alexa Fluor 488 (1:300) and Alexa Fluor 555 (1:300) secondary antibodies (Invitrogen). Tissue stained with secondary antibodies only served as controls. Images were taken using a Zeiss LSM 710 META confocal microscope with ZEN 2010 software (Carl Zeiss) using a 63x/NA 1.4 objective at an optical slice thickness of 1.0 μm.

**Table 1.** Antibody Table

Peptide/Protein Target	Antigen Sequence (if known)	Name of Antibody	Manufacturer, Catalog Number, and/or Name of Individual Providing the Antibody	Species Raised in; Monoclonal or Polyclonal	Dilution Used
SST	Synthetic cyclin (1–14) SST conjugated to bovine thyroglobulin	Rabbit anti-SST	Dako, A0566	Rabbit polyclonal	1:1000
GFP	Recombinant full-length GFP made in *Escherichia coli*	Anti-GFP antibody	Abcam, Ab5450	Goat polyclonal	1:1000

### Primary stomach epithelium mixed cell culture

The antrum and corpus of the stomach was separated and flushed with ice-cold PBS. The tissue was treated with 0.25-mg/mL collagenase XI. Digested tissue was collected via centrifugation at 100*g*, and isolated epithelial glands were resuspended in DMEM (25 mmol/L glucose) supplemented with 10% (vol/vol) fetal bovine serum, 2 mmol/L L-glutamine, 100-U/mL penicillin, and 100-μg/mL streptomycin. This tissue suspension was plated on matrigel (BD Biosciences-coated 24-well plates or 35-mm glass-bottom dishes; MatTek Corp), and incubated at 37°C under 5% CO_2_. For secretion assays, tissue from 3 mice was combined into the same plate. This was performed by digesting each stomach separately, then combining the pelleted glands from all 3 stomachs before resuspending in 12.5 mL of culture medium. A total of 500 μL of this resuspension was deposited into each well of 1 24-well plate.

### SST secretion

Secretion assays were conducted on cultures 1 day after plating tissue on 24-well plates. Cultures were incubated with test reagents made up in standard bath solution containing 0.1% (wt/vol) fatty acid-free BSA and 10 mmol/L glucose for 2 hours at 37°C. Hormone content in secretion samples was assayed using an ELISA (SST ultrasensitive ELISA kit; Phoenix Pharmaceuticals, Inc). Due to variations in epithelial gland size and the number of cells per gland, the number of cells seeded into each well is unknown. To account for variation in D-cell number between experimental replicates, the amount of SST measured in each well per 24-well plate was normalized to the average amount of SST measured in wells treated with control conditions (3–4 wells per 24-well plate) measured in parallel in the same plate on the same day, giving the fold change in SST secretion. The average secretion of SST in control conditions across all experiments was 27.4 ± 1.8 pg per well. Each assay condition was repeated in at least 3 wells per 24-well plate in at least 3 separate 24-well plates, each established from 3 murine stomachs.

### Calcium imaging

Stomach epithelial cells were imaged 1–2 days after plating. Cells were loaded with 5 μmol/L Fura-2 AM (Invitrogen) in standard bath solution containing 300 μmol/L eserine, 0.01% (vol/vol) pluronic, and 10 mmol/L glucose for 30 minutes at 37°C. Experiments were performed using a Hamamatsu Orca-ER digital camera (Cairn Research) attached to an Olympus IX71 inverted fluorescent microscope (Southall) with a 40× oil-immersion objective. Gastric D-cells were identified by their tdRFP expression when excited at 555/10 nm and collecting the emission with a 570 long pass dichroic mirror. To measure intracellular Ca^2+^ levels, cells were excited at 340/16 and 380/8 nm (emission 510/80), and images were taken every 2 seconds using a 75-W xenon arc lamp. Excitation and recording were controlled using Metafluor software (version 7.6.5.0; MDS Analytical Technologies). Recordings were background subtracted and represented as the 340/380 nm ratio. Primary cultures from mice expressing the genetically encoded Ca^2+^ indicator, GCaMP3, were imaged as above, except instead of loading with fura 2, cells were allowed to equilibrate to room temperature for 10 minutes in standard bath solution before imaging. To measure intracellular Ca^2+^ levels, cells were excited at 488/8 nm, and images were taken every 2 seconds using a 75-W xenon arc lamp. Emission was collected using a 510-nm long-pass filter. Recordings were background subtracted and represented as the 488-nm fluorescence intensity. Test reagents were perfused in standard bath solution at a rate of approximately 1 mL/min. Average fluorescence ratios/intensities were calculated over 10-second time windows for the entirety of the experiment. Responses to test reagents were calculated by determining the peak 340/380 (Fura-2) ratio or peak 488 fluorescence (GCaMP3) during perfusion of the test reagent divided by the average of the baseline taken before and after test reagent application to give a fold change value. Cells were included in the analysis if they responded to 30 mmol/L KCl.

### Stomach epithelial cell isolation for flow cytometry

After removal of the fundus, gastric epithelial cells were isolated as previously described by Schepp et al (32). Cells were immediately sorted by flow cytometry using a BD Influx cell sorter (BD Biosciences) equipped with a 488 laser for detection of EYFP. Cells were collected into RLT lysis buffer (QIAGEN) and frozen on dry ice.

### RNA extraction and quantitative RT-PCR (qPCR)

Total RNA was extracted using an RNeasy Micro kit (QIAGEN), according to the manufacturer's protocol. qPCR was performed with a 7900 HT Fast Real-Time PCR system (Applied Biosystems). The PCR reaction mix consisted of first-strand cDNA template, primer pairs, 6-carboxyfluorescein/quencher probes (Bioresearch Technologies and Applied Biosystems), and PCR Master mix (Applied Biosystems), and was amplified for 40 cycles. No-template controls were included for all primers. Expression of selected targets was compared with that of *Actb* measured on the same sample in parallel on the same plate, giving a CT difference (ΔCT) for *Actb* minus the test gene. Mean, SE, and statistics were performed on the ΔCT data and only converted to relative expression levels (2̂ΔCT) for presentation in the figures.

### RNA sequencing

Total RNA was extracted using an RNeasy Micro Plus kit (QIAGEN) according to the manufacturer's instructions. The quality of the RNA extracted was checked using a Bioanalyser RNA Pico kit (Agilent) with RNA integrity number values between 7.2 and 9.4. RNA was amplified using Ovation RNA-seq System V2 (NuGEN), whereby 2.5 ng of RNA for each sample were used (note 4 replicates were used each for D-cells and control cells, totaling 8 samples). To prepare the RNAseq library, the amplified cDNA (3 μg per sample) was fragmented to 200 bp using the Bioruptor Sonicator (Diagenode), and barcode ligation and end repair were achieved using the Ovation Rapid DR Library System (NuGEN). The barcoded libraries were combined and sent for SE50 sequencing using an Illumina HiSeq 2500 system at the Genomics Core Facility, Cancer Research UK Cambridge Institute.

### Data analysis

All statistical analysis was conducted using Microsoft Excel and GraphPad Prism 5.0 (GraphPad Software, Inc). Statistical significance was calculated using a Student's single-sample or 2-sample *t* test or via ANOVA with either a Dunnett's or a Bonferroni post hoc test, as appropriate. The threshold for significance was set as *P* < .05. Sequence reads were demultiplexed using the Casava pipeline (Illumina) and then aligned to the mouse genome (GRCm38) using Tophat version 2.0.11 (http://ccb.jhu.edu/software/tophat/index.shtml). Differential gene expression was determined using Cufflinks version 2.2.1 (http://cole-trapnell-lab.github.io/cufflinks/).

## Results

### Generation and validation of SST-Cre mice

**Figure 1.**
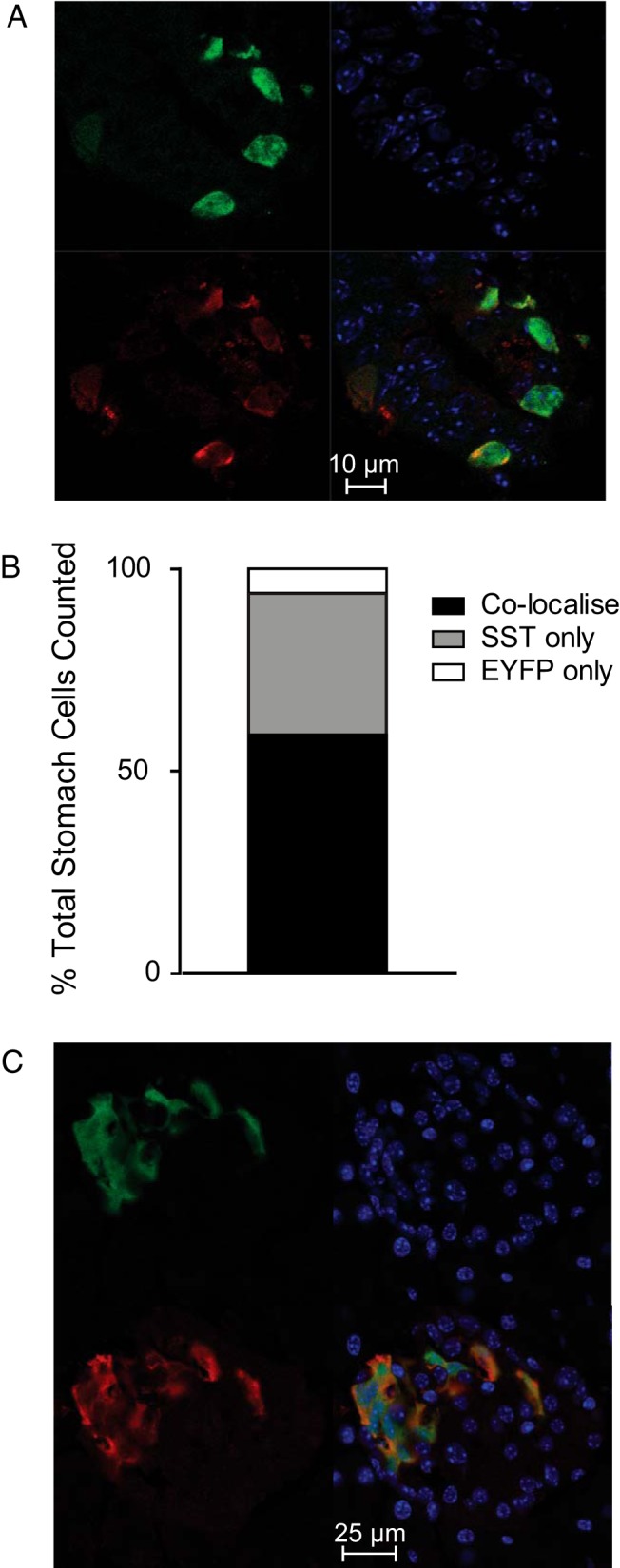
Confirmation of cell-specific transgene expression. A, Corpus/antral tissue isolated from transgenic mice expressing EYFP under the control of the *Sst* promoter was fixed, sliced, and stained for EYFP (green) and SST (red). Blue represents Hoechst staining. B, We counted 158 cells that were stained for EYFP and/or SST. The number of cells that were stained for SST only (gray), EYFP only (white), or both (black) are expressed as a percentage of all fluorescent cells counted. These data were obtained from 4 sections of whole stomach from one transgenic mouse. C, To investigate transgene expression in other areas known to express SST, slices of whole pancreas from mice expressing EYFP under the control of the *Sst* promoter were stained for EYFP (green) and SST (red). Blue represents Hoechst staining.

SST-Cre/ROSA26^tdRFP/EYFP^ mice were found to exhibit EYFP fluorescence that, in the stomach, was restricted to a subpopulation of cells. Cre recombinase efficiency in the stomach was assessed by quantifying the number of SST producing cells coexpressing EYFP via immunohistochemistry. In whole-stomach slices, costaining with antibodies against SST and EYFP revealed that 59% of SST-positive cells stained for EYFP and 91% of EYFP labeled cells were positive for SST (Figure 1, A and B, and Supplemental Figure 1, B and C). The percentage colocalization was also assessed in primary gastric epithelial cultures from SST-Cre/ROSA26^tdRFP^ mice, revealing that 80% of SST-positive cells contained RFP and 91% of RFP-expressing cells were positive for SST (225 cells counted in total, from 2 separate preparations, each containing pooled tissue from 4 transgenic mice) (Supplemental Figure 1D). This confirmed that transgene expression was largely restricted to cells producing SST, but indicated that Cre-recombinase action was not fully efficient and labeled 60%–80% of D-cells.

Slices of whole pancreas were also examined for transgene expression (Figure 1C). Staining for EYFP was largely isolated to SST-producing δ-cells. Importantly, no labeling was detected in the surrounding exocrine tissue where SST expression is known not to occur (Supplemental Figure 1E) (33).

### FACS purification and expression analysis of D-cells

SST-Cre/ROSA26^EYFP^ mice were used to purify D-cells by flow cytometry from epithelial digests of the gastric antrum and corpus based on their cell-specific fluorescence and low side scatter (Supplemental Figure 2). Control nonfluorescent cell populations were collected in parallel using stringent forward and side scatter gating that in pilot FACS analysis experiments was found to exclude cells that were YFP positive but *SST* negative. qPCR analysis of the relative expression of *Sst* in the EYFP positive and EYFP negative populations revealed a 690-fold (*P* < .001) enrichment of *Sst* message in the EYFP positive population.

**Figure 2.**
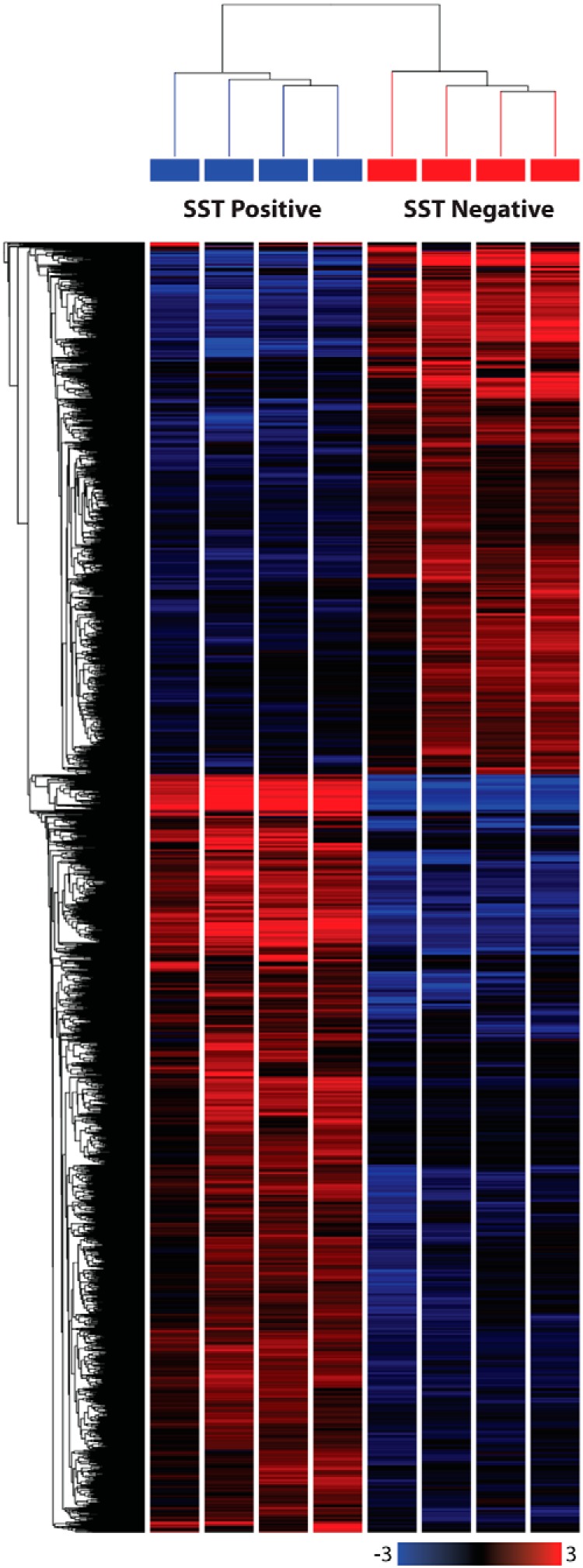
Heatmap showing differential expression of genes between SST positive and negative gastric epithelial cells. Single-cell digests of gastric mucosa from the antrum and corpus of SST-Cre x ROSA26^EYFP/EYFP^ mice were passed through a FACS sorter and gastric D-cells were purified based on their green fluorescence. RNA from 4 EYFP-positive and 4 EYFP-negative cell samples was sequenced using SE50 sequencing. Reads were aligned to the GRCm38 mouse genome using Tophat version 2.0.11. Differential gene expression was determined using Cufflinks version 2.2.1. The heatmap was generated using the following selection criteria: fold change of more than 2, false discovery rate 0.1, and FPKM values of more than 1, and was subjected to hierarchical clustering based on Euclidean distance and average linkage. A total of 3143 genes were differentially expressed. More information on these genes can be found in Supplemental Dataset 1.

**Figure 3.**
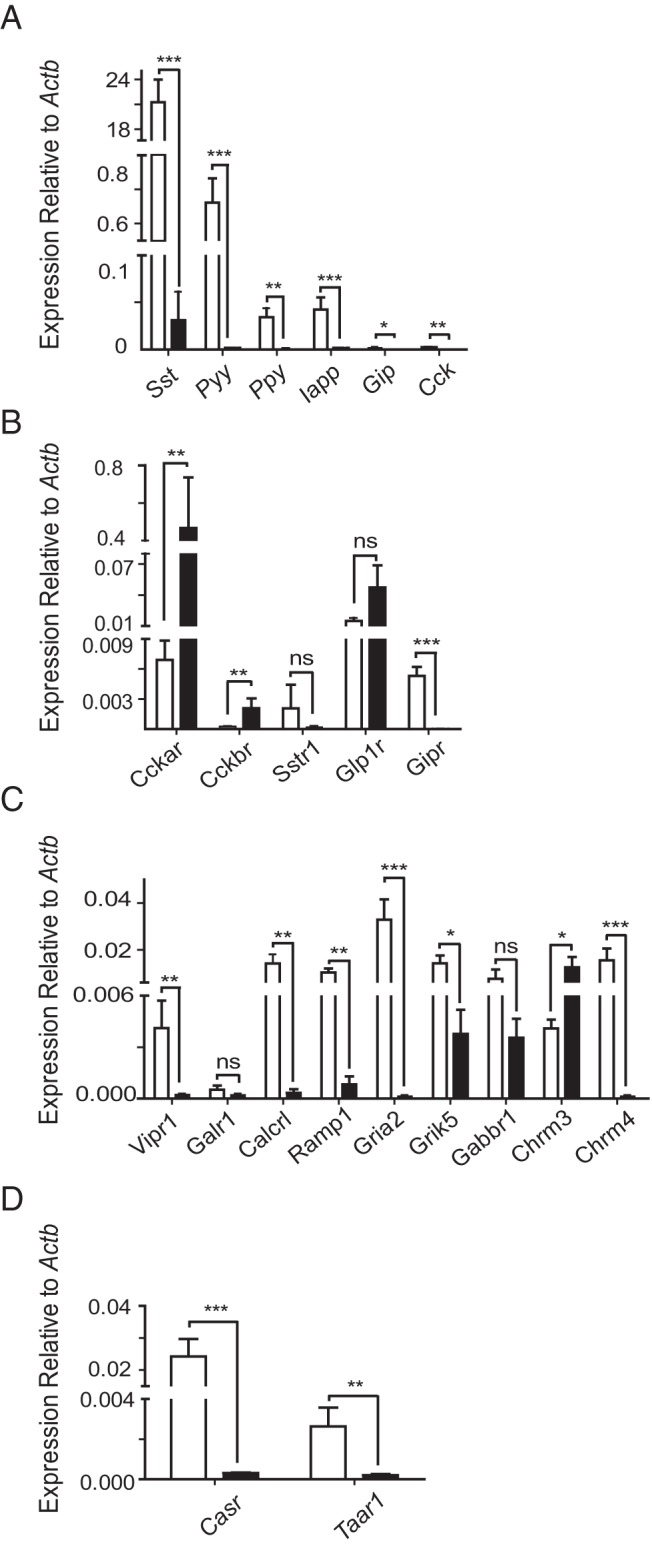
Gene expression in SST-positive and SST-negative cells measured by qPCR. Histograms showing relative gene expression of peptide hormones (A), peptide hormone receptors (B), neurotransmitter receptors (C), and nutrient receptors (D) in SST-positive (white bars) and SST-negative (black bars) cells. Expression was analyzed by qPCR and compared with that of *Actb* in the same sample. Each column represents 3 samples from separate mice. Samples where target genes were undetected were assigned Ct values of 40. Data are presented as the geometric mean, with error bars calculated from log(base2) data. Significance comparisons between SST-positive and SST-negative cells were calculated by Student's *t* test performed on the nontransformed ΔCT data; ns, not significant; *, *P* < .05; **, *P* < .01; ***, *P* < .001.

We next performed RNA sequencing (RNA-seq) analysis of these isolated D-cells and control populations to identify hormones, GPCRs, and other receptors that might underlie the behavior and modulation of gastric D-cells. The average total number of reads per sample was 11 million, with an average of 9.4 million reads mapping to the mouse genome, thereby resulting in a mapping efficiency of 85.7%. Using a fold change cut-off of 2, a false discovery rate of 0.1, and a sensitivity threshold for expression of more than 1 fragment per kilobase per million mapped reads (FPKM), RNA-seq analysis found that 3143 genes were differentially expressed between *Sst*-positive and *Sst*-negative cell populations (Figure 2 and Supplemental Dataset 1). Expression of key targets selected from this list was confirmed by qPCR (Figure 3 and Supplemental Figure 4).

RNA-seq analysis revealed that transcript levels for *Pyy*, *Gip*, *Ppy*, *Cck*, and *Iapp* (amylin) were all enriched in D-cells over neighboring epithelial cells (Table 2), indicating a possible coproduction of these peptides with SST, and potential heterogeneity within the SST-expressing cell population. D-cell expression of these hormones was confirmed by qPCR, although even the most abundant alternative hormonal transcript (*Pyy*) was found at approximately 30-fold lower levels than *Sst* (Figure 3A).

**Table 2.** RNA Sequencing Analysis of Select Genes Expressed in Gastric D-Cells and Neighboring Gastric Epithelial Cells

	SST Positive (FPKM)	SST Negative (FPKM)
Coexpressed hormones		
*Sst*	82 900 ± 12 200	242 ± 34
*Pyy*	5260 ± 963	17.2 ± 4.9
*Ppy*	114 ± 18	0.55 ± 0.20
*Iapp*	38.8 ± 6.1	0.68 ± 0.59
*Gip*	67.7 ± 18.3	0.24 ± 0.14
*Cck*	37.2 ± 10.1	0.52 ± 0.22
Hormone receptors		
*Glp1r*	8.23 ± 1.35	11.7 ± 2.7
*Gipr*	1.96 ± 0.32	0.02 ± 0.02
*Cckar*	3.53 ± 0.41	23.5 ± 6.7
*Cckbr*	0.19 ± 0.09	1.84 ± 0.72
*Sstr1*	1.93 ± 0.84	0.02 ± 0.02
*Sstr2*	1.01 ± 0.17	1.81 ± 1.22
*Sstr3*	2.99 ± 0.62	0 ± 0
*Sstr4*	0 ± 0	0 ± 0
*Sstr5*	0.02 ± 0.01	0.01 ± 0.01
Neurotransmitter receptors		
*Vipr1*	2.00 ± 1.47	0.07 ± 0.07
*Calcrl*	50.9 ± 10.2	0.76 ± 0.25
*Ramp1*	22.2 ± 2.3	0.68 ± 0.08
*Galr1*	1.62 ± 0.33	0.07 ± 0.05
*Nmbr* (*Bb1*)	0.07 ± 0.07	0 ± 0
*Grpr* (*Bb2*)	0.09 ± 0.07	0 ± 0
*Brs3* (*Bb3*)	0 ± 0	0 ± 0
*Gria2*	6.00 ± 1.07	0.02 ± 0.02
*Grik5*	1.12 ± 0.38	0.07 ± 0.04
*Chrm1*	0.12 ± 0.05	0.80 ± 0.13
*Chrm2*	0.04 ± 0.03	0 ± 0
*Chrm3*	0.43 ± 0.06	0.32 ± 0.12
*Chrm4*	9.80 ± 2.01	0.03 ± 0.03
*Chrm5*	0 ± 0	0.04 ± 0.04
*Hrh1*	0 ± 0	0.01 ± 0.01
*Hrh2*	0.47 ± 0.11	0.76 ± 0.23
*Hrh3*	0.27 ± 0.10	0 ± 0
*Hrh4*	0 ± 0	0 ± 0
*Gabbr1*	11.8 ± 3.1	0.97 ± 0.43
*Gabra1*	3.69 ± 0.44	0.03 ± 0.01
*Gabrb3*	35.3 ± 3.2	0.48 ± 0.16
Nutrient-derived factor receptors		
*Casr*	7.32 ± 1.69	0.03 ± 0.03
*Taar1*	6.81 ± 0.85	0.04 ± 0.04
*Gpr92/Lpar5*	2.85 ± 1.12	2.08 ± 1.03
*Gprc6a*	0.12 ± 0.06	0 ± 0
*Tas1r1*	0.01 ± 0.01	0.01 ± 0.01

Data are averaged FPKM values for genes of interest. Data are representative of 4 samples each from 4 separate mice for SST positive and negative cells.

RNA-seq analysis identified a number of gut hormone receptor transcripts with FPKM values of more than 1 in D-cells, specifically receptors for GLP-1 (*Glp1r*), GIP (*Gipr*), CCK (*Cckar*), and the SST receptors *Sstr1*, *Sstr2*, and *Sstr3*, whereas D-cell CCK-B receptor (*Cckbr*) transcripts were very few. qPCR data confirmed these expression patterns for *Glp1r*, *Gipr*, *Cckar*, and *Cckbr*. Of note, the expression of *Gipr* was largely D-cell specific, with D-cell expression levels nearly 100-fold higher than in neighboring cells (*P* < .001). The finding that *Cckbr* was barely detectable in D-cells indicates that *Cckar* is the dominant CCK receptor family member in these cells (Figure 3B).

The expression of receptors for prominent neurotransmitters in the gut was also examined. Receptors for the neuropeptides VIP and PACAP (*Vipr1*), CGRP (*Calcrl* and *Ramp1*), and galanin (*Galr1*) were detected and enriched in D-cells. qPCR confirmed that D-cells expressed significantly higher levels of *Vipr1* (*P* < .01), *Calcrl* (*P* < .01), and *Ramp1* (*P* < .01) than non-D-cells. In contrast, members of the bombesin receptor family (*Bb1–3*) exhibited FPKM values of less than 0.2, suggesting they are relatively poorly expressed. Of the muscarinic receptor family, we detected high, D-cell-specific expression of *Chrm4* (M_4_) and low expression of *Chrm3* (M_3_), whereas levels of *Chrm1*, *Chrm2*, and *Chrm5* in D-cells were very low. Among the glutamate receptor family, we observed high D-cell-specific expression of the ionotropic glutamate receptor, *Gria2*, and moderate to low levels of the kainite receptor *Grik5* in both D-cells and their neighbors. Expression of histamine receptor family members (*Hrh*) in D-cells was also very low (Table 2). Again, qPCR analysis revealed good correlation with the RNA-seq findings: expression of *Gria2*, *Grik5*, *Chrm3*, and *Chrm4* was confirmed, and levels of *Gria2*, *Grik5*, and *Chrm4* were significantly higher in D-cells than their neighbors (Figure 3C).

Because SST release is regulated in vivo by luminal protein digestion, we examined D-cell expression of candidate receptors for oligopeptides and amino acids. One of the most highly enriched D-cell transcripts, *Casr*, encodes the calcium-sensing receptor (CASR), a GPCR capable of sensing extracellular Ca^2+^, aromatic amino acids, and oligopeptides (34, 35). The lysophosphatidic acid receptor 5, LPAR5 (GPR92/93), previously implicated in peptone sensing by EECs, was also identified in D-cells by RNA-seq. Of the candidate amino acid sensors, we did not find high levels of any members of the metabotropic glutamate receptor family, the umami receptor subunit (*Tas1r1*) or *Gprc6a*. D-cells did, however, express high levels of the trace amine receptor *Taar1*, a GPCR known to bind trace amines, products of the decarboxylation of amino acids found in fermented foodstuffs (Table 2) (36). By qPCR analysis, both *Taar1* and *Casr* were confirmed to be D-cell specific (*P* < .01 and *P* < .001, respectively): expression of *Casr* was almost 80-fold greater in D-cells vs neighboring cells, whereas *Taar1* expression was 13-fold higher in D-cells (Figure 3D).

### Identification of stimuli affecting SST release and D-cell intracellular Ca^2+^ activation

In order to establish a functional role for the receptors found to be enriched in RNA-seq and qPCR analyses, primary cultures of the mouse gastric antrum epithelium were maintained for approximately 24 hours and then stimulated for 2 hours with candidate stimuli.

**Figure 4.**
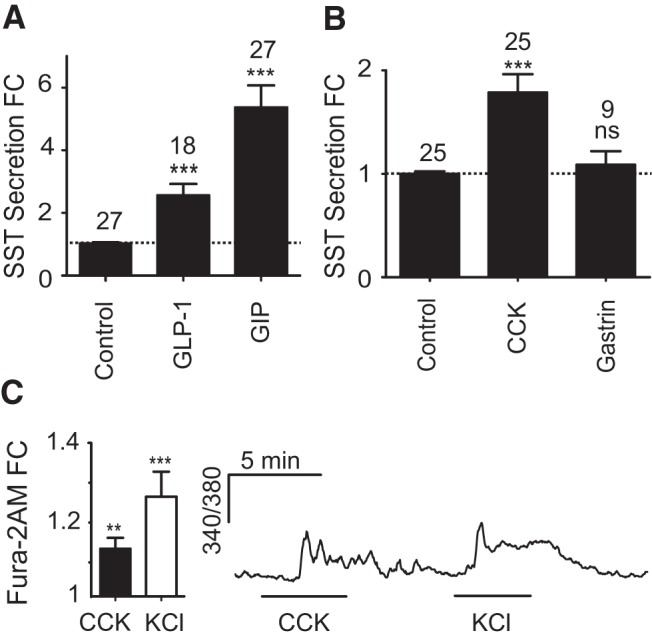
Gut hormone-regulated SST secretion from primary gastric epithelial cultures. Mixed gastric epithelial cultures from the corpus and antrum were incubated for 2 hours in bath solution containing the regulatory gut peptides, GIP and GLP-1 (both 100 nmol/L) (A) and CCK and gastrin (both 100 nmol/L) (B). SST was measured in the supernatant fraction and is expressed relative to basal secretion measured in parallel on the same day (control). FC is fold change compared with control (indicated by the dashed line); n numbers for each condition are represented above each bar. Error bars represent 1 SE, and significance is shown analyzed by ANOVA with a post hoc Dunnett modification; ***, *P* < .001. C, Gastric epithelium from transgenic mice expressing tdRFP under the control of the *Sst* promoter was cultured for 24 hours and loaded with Fura-2 AM. D-cells identified by tdRFP fluorescence were excited at 340 nm, and the 340/380 nm fluorescence ratio was recorded (reflecting changes in [Ca^2+^]_i_). Cells were perfused with standard bath solution containing either 100 nmol/L CCK, or 30 mmol/L KCl, as indicated. Mean changes in calcium after the addition of CCK or KCl were calculated by normalizing the peak response to the 340/380 ratio at baseline before and after the stimulus (fold change [FC]). Seven out of 11 cells responded to CCK. Data represent the mean and SE of responding cells. Significance compared with baseline was calculated using a single Student's *t* test; **, *P* < .01; ***, *P* < .001.

The incretin peptides, GIP and GLP-1 (100 nmol/L each), evoked significant increases in SST secretion (5.4-fold, *P* < .001; 2.6-fold, *P* < .001, respectively) (Figure 4A), and CCK enhanced SST secretion 1.8-fold (*P* < .001). In agreement with the gene expression data, gastrin failed to stimulate SST release from primary cultures (Figure 4B).

CCK-A and CCK-B are G_q_-coupled receptors (37), and their activation should therefore trigger phospholipase-C-dependent release of intracellular Ca^2+^ stores. By Fura-2 imaging, Ca^2+^ was found to be elevated by CCK in 7 out of 11 D-cells (mean 1.12-fold increase in fluorescence ratio, *P* < .01, for responding cells only) (Figure 4C), indicating direct regulation of D-cells by CCK. In agreement with the gene expression and secretion assay data, 100 nmol/L gastrin failed to elevate D-cell Ca^2+^ (data not shown).

**Figure 5.**
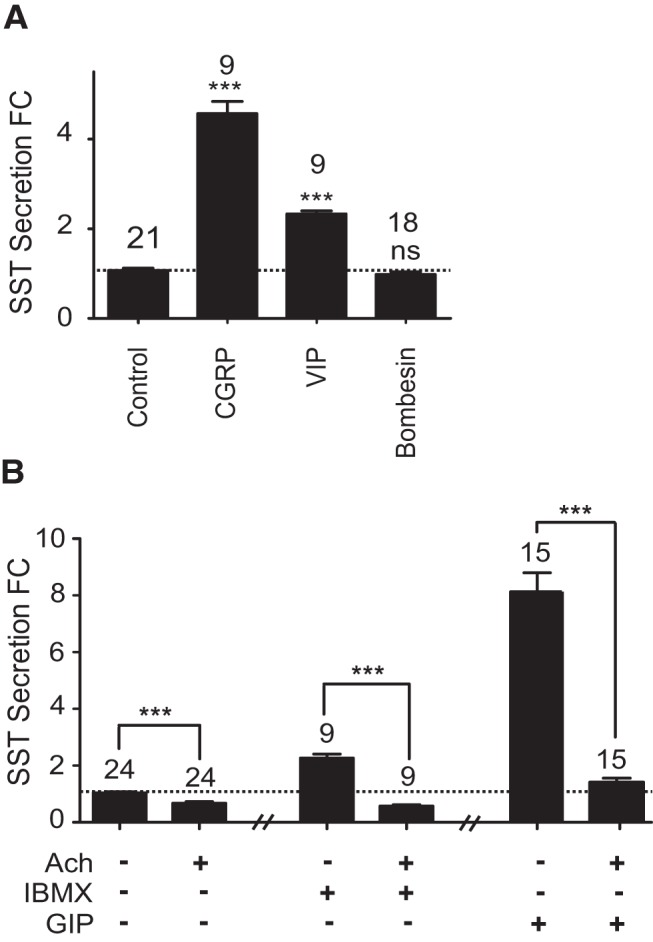
Neurotransmitter-regulated SST secretion from primary gastric epithelial cultures. Mixed gastric epithelial cultures from the corpus and antrum were incubated for 2 hours in bath solution containing the stimulatory neurotransmitters CGRP, VIP, and bombesin (all 100 nmol/L) (A) or acetylcholine (Ach) (10 μmol/L) with or without IBMX or GIP (100 μmol/L or 100 nmol/L, respectively) (B). SST was measured in the supernatant fraction and is expressed relative to basal secretion measured in parallel on the same day (control). FC is fold change compared with control (indicated by the dashed line); n numbers for each condition are represented above each bar. In B, the effect of Ach on IBMX and GIP was assayed in different preparations. The control data accompanying both Ach on IBMX and Ach on GIP experiments have been combined. Error bars represent 1 SE, and significance is shown analyzed by ANOVA with a post hoc Dunnett or Bonferroni modification where appropriate; ***, *P* < .001; ns, not significant.

Strong SST secretory responses were observed after application of a number of neurotransmitters. CGRP (100 nmol/L) stimulated secretion 4.6-fold (*P* < .001), and VIP (100 nmol/L) elicited a 2.3-fold increase in SST release (*P* < .001). No significant response was observed with bombesin, an analog of gastrin-releasing peptide (GRP), in accordance with the lack of expression of bombesin receptors in the RNA-seq data (Figure 5A). Acetylcholine (10 μmol/L) proved to be a potent inhibitory influence on SST release. When applied in basal conditions, acetylcholine decreased SST levels by approximately 45% (*P* < .001), and in the presence of 100 μmol/L 3-isobutyl-1-methylxanthine (IBMX), acetylcholine decreased SST secretion to below basal levels (*P* < .001) (Figure 5B). Acetylcholine when applied in the presence of 100 nmol/L GIP also decreased SST secretion to basal levels (*P* < .001) (Figure 5B).

**Figure 6.**
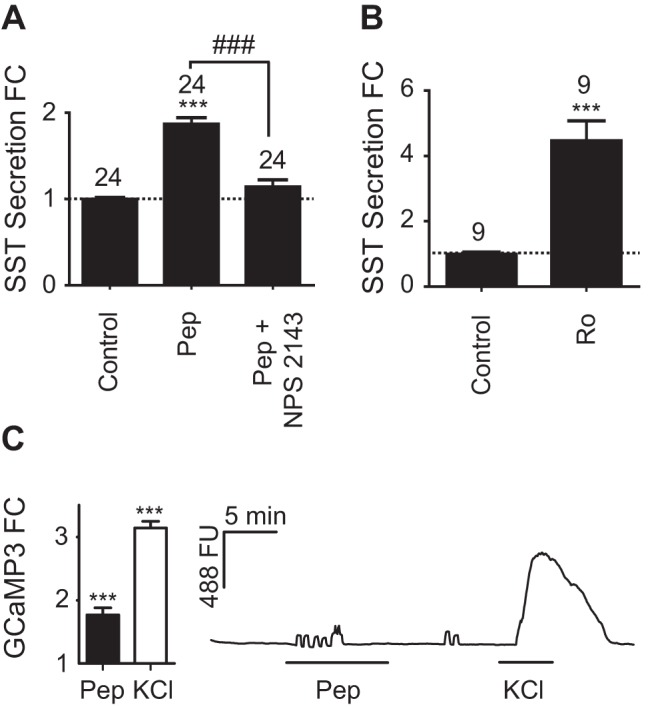
Nutrient-regulated SST secretion from primary gastric epithelial cultures. Mixed gastric epithelial cultures from the corpus and antrum were incubated for 2 hours in bath solution containing peptones (Pep) (0.5% wt/vol) with or without NPS 2143 (5 μmol/L) (A), or Ro5166017 (Ro) (50 nmol/L) (B). SST was measured in the supernatant fraction and is expressed relative to basal secretion measured in parallel on the same day (control). FC is fold change compared with control (indicated by the dashed line); n numbers for each condition are represented above each bar. Error bars represent 1 SE, and significance is shown analyzed by ANOVA with a post hoc Bonferroni modification or Student's *t* test, where appropriate; *** or ###, *P* < .001. C, Gastric epithelium from transgenic mice expressing tdRFP and the genetically encoded calcium sensor, GCaMP3, under the control of the *Sst* promoter was cultured for 24 hours before imaging. D-cells identified by tdRFP and GCaMP3 fluorescence were excited at 488 nm, and the GCaMP3 fluorescence (FU) was recorded (reflecting changes in [Ca^2+^]_i_). Cells were perfused with standard bath solution containing either 0.5% wt/vol Pep or 30 mmol/L KCl, as indicated. Mean changes in calcium after the addition of Pep or KCl were calculated by normalizing the peak response to the 488 fluorescence at baseline before and after the stimulus (fold change [FC]). Twelve out of 38 cells responded to Pep. Data represent the mean and SE of responding cells. Significance compared with baseline was calculated using a single Student's *t* test; ***, *P* < .001.

Peptone (0.5% wt/vol), an enzymatic meat hydrolysate, elicited a 1.9-fold increase in SST secretion (*P* < .001) that was significantly inhibited by the CASR antagonist NPS2143 (5 μmol/L, *P* < .001) (Figure 6A). We also examined the potential effect of trace amines, a subgroup of biogenic amines derived from amino acid metabolites (36). Agonism of trace amine-associated receptor 1 (TAAR1) using 50 nmol/L Ro5166017 (38) elicited a strong 4.5-fold increase in SST secretion (*P* < .001) (Figure 6B).

To examine the putative direct effect of peptones on D-cells via the G_q_-coupled CASR (39), we employed a novel triple transgenic mouse strain, SST-Cre/ROSA26^tdRFP/GCaMP3^ expressing tdRFP and the genetically encoded calcium indicator GCaMP3 specifically in the D-cell population. In pilot experiments in which we recorded simultaneously from fura 2 and GCaMP3, similar responses in gastric D-cells were recorded using the 2 techniques (Supplemental Figure 5). Further exploration of the D-cell Ca^2+^ response to peptone (0.5% wt/vol) using GCaMP3 imaging revealed that 12/38 cells responded with an increase in intracellular Ca^2+^ (1.74-fold increase in fluorescence, *P* < .001) (Figure 6C).

## Discussion

### Cre-mediated labeling of SST-producing cells

Our use here of SST-Cre mice has enabled the identification and single-cell characterization of gastric D-cells. Immunohistochemical analysis confirmed that the SST-Cre line correctly labeled D-cells in the gastric mucosa and endocrine pancreas. We found the Cre penetrance of this mouse line did not enable the labeling of all gastric SST-producing cells, in accordance with a previous study reporting 80% efficiency of Cre-dependent reporter expression in the pancreas (40). Despite moderate labeling efficiency, this SST-Cre mouse line is the first to permit FACS-based purification of gastric D-cells for transcriptomic analysis as well as the real-time imaging of D-cell Ca^2+^ responses.

A caveat for the use of this mouse line in transcriptomic analysis of FACS-purified SST positive and negative populations is that 1) not all SST-producing cells were labeled by the fluorescent reporter and 2) a small subset of fluorescently labeled cells did not stain for SST. The impact of the former is that we might not have captured all D-cell RNAs if the fluorescent labeling of SST-positive cells was nonrandom. To reduce the impact of the latter, we performed early pilot FACS analysis experiments using fixed and permeabilized stomach epithelial cells immunohistochemically double stained for EYFP and SST, which revealed that SST-positive cells could be distinguished from EYFP-positive/SST-negative cells by their characteristic low side and forward scatter. A low forward/side scatter gate was therefore used during FACS sorting to optimize the purity of the D-cell population and resulted in nearly 700-fold enrichment for SST expression in positive compared with negative populations. The negative population is predicted to contain a mixture of stomach epithelial cell types, including chief and parietal cells, G-cells, and enterochromaffin-like cells, but due to the constrained forward and side scatter gates used to collect SST-negative cells, not all stomach epithelial cell types may be represented in the negative population.

Consistent with previous reports showing that many EECs express a variety of regulatory hormones (41), D-cells also expressed transcripts for *Pyy*, *Ppy*, *Iapp*, *Gip*, and *Cck*, albeit at low levels. Although our data do not indicate whether these transcripts arise from all D-cells or only subpopulations, previous studies looking at PYY-associated immunoreactivity in the gut reported that a subset of D-cells in the gastric mucosa costained for PYY (42).

### Hormonal regulation of D-cells

In our primary stomach mixed epithelial cultures, both GLP-1 and GIP enhanced SST release, consistent with a previous study using perfused rat stomachs that reported a stimulation of SST secretion by GLP-1 (26). The perfusion experiments similarly concluded that this stimulation was likely to be a direct effect on enteroendocrine D-cells rather than mediated by the stimulation of SST-producing nerves, because tetrodotoxin had no effect on GLP-1-stimulated SST release (26). Studies in perfused rat stomach also found that GIP infusion stimulated SST release (29), which was abolished by acetylcholine or stimulation of the vagus nerve (29), mirroring our secretion results. Although we cannot rule out the role of paracrine signaling in our primary mixed culture secretion assays, taken together with our D-cell mRNA expression data, these results support the idea that incretin hormones directly stimulate D-cells. If gastric SST is capable of reaching the more distal L- and K-cell populations, this in turn might result in a feedback inhibition of GLP-1 and GIP release. A negative feedback loop involving SST has also been suggested in previous studies: in humans, treatment with GLP-1 mimetics or dipeptidyl peptidase-4 inhibitors results in lower endogenous GLP-1 release (43), and in pig perfused intestine, antagonists of SST receptors increased GLP-1 secretion (44).

Both incretins have been found to decrease gastric acid production (45), and in perfused rat stomachs addition of GLP-1 led to a concomitant drop in gastrin production alongside SST stimulation. This combined with our secretion data could suggest that the inhibitory effect of incretins on gastric acid secretion is partly mediated by SST (26).

CCK and gastrin share an amidated carboxy-terminal pentapeptide sequence (46) and bind the same CCK-A and CCK-B receptors, with gastrin preferentially binding CCK-B (47–49). Both CCK-A and CCK-B are present in the GI tract, including mucosal cells (46, 47, 50). Although previous literature suggested that both gastrin and CCK elicit SST release from the stomach (47, 51–54), in our primary epithelial cultures, CCK but not gastrin increased SST secretion and intracellular Ca^2+^ in D-cells. These functional results were supported by our gene expression data indicating the presence of CCK-A but not CCK-B receptors in D-cells. Our results are consistent with a number of other previous findings. Immunostaining in human gastric samples, for example, confirmed the presence of CCK-A but not CCK-B receptors in SST-positive cells (46), and CCK has been reported to act through CCK-A receptors in cultures of canine fundic D-cells, leading to an increase in SST secretion (28, 55, 56). Studies suggesting that gastrin stimulates SST release via a negative feedback loop involving CCK-B receptors, have mostly been performed in whole tissues, and could not distinguish whether the CCK-B receptors were located directly on D-cells (57). Our results rather suggest that any effects of gastrin on SST secretion are likely to be indirect, involving CCK-B receptors on a different cell type.

### Neural regulation of D-cells

The stomach is densely innervated by the ENS, as well as processes of extrinsic neurons. In our secretion assays, several neurotransmitters proved to be effective stimuli or inhibitors of SST release, highlighting the potential importance of neural factors in regulating gastric physiology. CGRP, for example, was an effective stimulus for SST secretion, correlating with high D-cell expression of the CGRP receptor subunits *calcrl* and *ramp1*. These results support studies showing that activation of D-cells by low pH relies on the stimulation of CGRP-releasing peripheral sensory afferent nerve endings in the stomach (24). Indeed, a pathway involving CGRP release triggered by low pH could underlie the indirect elevation of SST release by gastrin.

Immunoreactivity for VIP can be found in nerve fibers interspersed between antral glands (58), and VIP has been shown to augment SST release and decrease gastric acid secretion in perfusion studies (58). In our mixed cultured epithelial cells, VIP stimulated SST secretion, and *vipr1* expression was significantly enriched in purified D-cells. Although not tested in our culture system, PACAP-38, produced by enteric neuronal cell bodies that innervate the gastric smooth muscle layer and mucosa, is also known to bind VIPR1 (59), and perfusion studies in mice have shown that PACAP-38 inhibits pentagastrin-triggered acid secretion via SST release (59). Our data suggest that VIPR1 located on D-cells could underlie these observed effects of PACAP-38 and VIP.

GRP is a neurotransmitter present in the mucosal nerve fibers of the fundus and antrum (60), and directly stimulates the release of gastrin from G-cells (61). Contrary to previous findings that GRP increased SST release from perfused rat stomach (62), we found no evidence for direct effects of GRP/bombesin on D-cells. Other studies have come to a similar conclusion (61, 63, 64), and some perfusion studies have suggested that the effects of bombesin/GRP on SST release are mainly indirect, perhaps involving the release of a noncholinergic stimulatory transmitter (57). Secretion assays in our mixed stomach epithelial cell cultures support the role of a neurotransmitter intermediary, because paracrine signaling between epithelial cells types might still be intact in our culture system, whereas neural regulation should be disrupted.

Acetylcholine proved to be a strong inhibitory factor in our secretion assays, consistent with extensive literature indicating that SST release is under cholinergic control. Until now it was not, however, clear which muscarinic receptors are responsible for direct cholinergic inhibition of D-cells. Of the muscarinic receptor family, only M_2_ and M_4_ are coupled to G_i_ proteins, whereas M_1_, M_3_, and M_5_ are predominantly G_q_ coupled. Our expression data suggest that *Chrm4* (M_4_) is the predominant inhibitory muscarinic receptor expressed in D-cells, although further functional assays are needed to confirm this.

Histamine is also purported to be a negative regulator of SST release acting through the H_3_ receptor, because perfusion studies in rat stomach using H_3_ antagonists showed an increase in SST release (65). In our RNA-seq data, however, transcripts for histamine receptors were not detected to an appreciable degree, consistent with previous immunohistochemical evidence that failed to detect H_3_ in gastric D-cells (66). The effect of histamine on SST release is therefore likely to be indirect.

### Nutrient regulation of D-cells

A variety of receptors and transporters have been suggested to play a role in the chemosensation of protein digestion products by EECs. Several studies have implicated the G_q_-coupled CASR in the detection of oligopeptides and aromatic amino acids (34, 35, 67, 68), and recent immunohistochemical analysis confirmed the presence of CASR in the gastric antrum, including in a subset of antral D-cells in mouse, swine, and human tissue (69). Our data show that *Casr* is highly and specifically expressed in D-cells and that the secretion of SST from cultured stomach epithelium in response to 0.5% peptones is sensitive to the specific CASR antagonist, NPS2143. These results point towards a role for CASR in D-cell peptone detection.

Other receptors that have been implicated in amino acid and oligopeptide detection include umami taste receptors (Tas1R1/3), metabotropic glutamate receptors, GPRC6A and GPR92 (LPAR5). Immunohistochemistry analysis has found that in addition to CASR, D-cells costain for GPR92 and GPRC6A (69, 70). Gastric cell fractionation studies found that SST-enriched fractions also expressed mRNAs for the metabotropic glutamate receptor (mGluR) subunits 2, 3, 4, and 7 (35, 71, 72). Although D-cell transcript levels for Tas1R1, GPRC6A and mGluRs in our RNA-seq analysis were very low, suggesting they are unlikely to be expressed in D-cells, *lpar5* was present in both D-cells and neighboring epithelial cells.

Although oligopeptides also stimulate gastrin release during early stages of protein breakdown and thereby promote gastric acid release, a rising concentration of protein digestion products in the gastric antrum could signal to D-cells that digestion has proceeded beyond a certain stage and that acid production can now be switched off.

### Trace amines

The trace amine receptor, TAAR1, was identified as a D-cell-specific GPCR in both RNA-seq and qPCR analysis. This G_s_-coupled receptor has been shown to recognize the trace amines tyramine and β-phenylethylamine, as well as other biogenic amines (38, 73). Although most studies addressing TAAR1 activity have focused on its role in neurotransmission, both qPCR and experiments exploring the binding sites of a radio-labeled 3-iodothyronamine, a potent natural agonist of TAAR1, have confirmed the presence of TAAR1 in the stomach (74). Because trace amines have been shown to be present at high levels in protein-rich substrates like meat and cheese, the ingestion of such foods might provide a plausible mechanism for the delivery of trace amines to the stomach lumen (75). The TAAR1-specific agonist Ro5166017 allowed for selective examination of TAAR1-mediated effects on D-cells, and we confirmed that TAAR1 activation robustly triggered SST release. Further work needs to be done to ascertain which naturally occurring trace amines affect SST secretion, but these findings have identified a novel mechanism by which food components could modulate gastric and intestinal function.

## Conclusion

The integration of neural, hormonal, and nutritional signals is critical for the coordination of digestion, nutrient absorption, and the secretion of regulatory peptides. SST provides an on/off switch for gastric acid secretion that has implications for the efficiency of digestion, as well as the protection against luminal acidosis. Our in depth transcriptomic and functional characterization of D-cells and their modulation by regulatory peptides, neurotransmitters, and dietary stimuli not only improves our understanding of gastric D-cells but promises to inform future studies focused on targeting D-cell activity.
